# Supplementary material for: Blood Immunophenotypes of Idiopathic Pulmonary Fibrosis: Relationship with Disease Severity and Progression
Source: Int J Mol Sci. 2023 Sep 7;24(18):13832. doi: 10.3390/ijms241813832 (PMC10531459; doi:10.3390/ijms241813832)
Supplement: Supplementary file 1 [file ijms-24-13832-s001.zip › ijms-2550704-supplementary.pdf]

Blood Immunophenotypes of Idiopathic Pulmonary Fibrosis: Relationship with Disease Severity and Progression

Nuria Mendoza [1,2,3], Sandra Casas-Recasens [1,2], Núria Olvera [1,2,4], Fernanda Hernandez-Gonzalez [1,3,5], Tamara Cruz [1,2], Núria Albacar [1,2,5], Xavier Alsina-Restoy [5], Alejandro Frino-Garcia [5], Gemma López-Saiz [5], Lucas Robres [2], Mauricio Rojas [6], Alvar Agustí [1,2,3,5], Jacobo Sellarés [1,2,5,†] and Rosa Faner [1,2,3,*,†]

[1] Institut d'Investigacions Biomediques August Pi i Sunyer (IDIBAPS), 08036 Barcelona, Spain; nmendoza@recerca.clinic.cat (N.M.); sacasas@recerca.clinic.cat (S.C.-R.); olvera@recerca.clinic.cat (N.O.); fhernandez@clinic.cat (F.H.-G.); cruz@recerca.clinic.cat (T.C.); albacar@clinic.cat (N.A.); aagusti@clinic.cat (A.A.), sellares@clinic.cat (J.S.)
[2] Centro de Investigación Biomédica en Red de Enfermedades Respiratorias (CIBERES), 28029, Madrid, Spain; lrobres@recerca.clinic.cat
[3] Biomedicine Department, Universitat de Barcelona, 08036 Barcelona, Spain
[4] Barcelona Supercomputing Center (BSC), 08034 Barcelona, Spain
[5] Respiratory Institute, Clinic Barcelona, 08036 Barcelona, Spain; xalsina@clinic.cat (X.A.-R.); adfrino@clinic.cat (A.F.-G.); gsaiz@recerca.clinic.cat (G.L.-S.)
[6] Department of Internal Medicine, The Ohio State University Wexner Medical Center, Columbus, OH 43210, USA; mauricio.rojas@osumc.edu
* Correspondence: rfaner@recerca.clinic.cat; Tel.: +34-93-227-1715; Fax: +34-93-227-1716
[†] These authors contributed equally to this work.

Online supplement: 314 words; 5 Tables; 3 Figures.

Supplementary tables

Table S1. Gating immunophenotypes explored in the flow cytometry analysis.

**Immune populations**	**Gating Phenotype (after $CD45^+$ cells/Single/Live)**	**% Reference population**
**Lymphocyte panel**		
Lymphocytes $CD3^+$	$CD45^+$ $CD3^+$	$CD45^+$ alive
$CD8^+$ T cells	$CD45^+$ $CD3^+$ $CD8^+$	$CD45^+$ $CD3^+$
$CD4^+$ T cells	$CD45^+$ $CD3^+$ $CD4^+$	$CD45^+$ $CD3^+$
Effector T cells	$CD45^+$ $CD3^+$ $CD45RA^+$ $CD197^-$	$CD4^+$/$CD8^+$ T cells
Effector memory T cells	$CD45^+$ $CD3^+$ $CD45RA^-$ $CD197^-$	$CD4^+$/$CD8^+$ T cells
Central memory T cells	$CD45^+$ $CD3^+$ $CD45RA^-$ $CD19^+$	$CD4^+$/$CD8^+$ T cells
Naive T cells	$CD45^+$ $CD3^+$$CD45RA^+$$CD197^+$	$CD4^+$/$CD8^+$ T cells
Th1 cells	$CD45^+$ $CD3^+$ $CD4^+$ $CD183^+$ $CD196^-$	$CD4^+$ T cells
Th17 cells	$CD45^+$ $CD3^+$ $CD4^+$ $CD183^-$ $CD196^+$	$CD4^+$ T cells
Regulatory T cells (Tregs)	$CD45^+$ $CD3^+$ $CD4^+$ $CD25^+$ $CD127^-$	$CD4^+$ T cells
HLA-DR^+ T cells	$CD45^+$ $CD3^+$ HLA-DR^+	$CD4^+$/$CD8^+$ T cells
Antigen experienced T cells	$CD45^+$ $CD3^+$ $CD28^-$	$CD4^+$/$CD8^+$ T cells
PD-1^+ cells	$CD45^+$ $CD3^+$ PD-1^+	$CD45^+$ $CD3^+$
NKT cells	$CD45^+$ $CD3^+$ $CD56^+$	Lymphocytes
B cells	$CD45^+$ $CD3^-$ $CD19^+$	Lymphocytes
NK cells	$CD45^+$ $CD3^-$ $CD56^+$	Lymphocytes
$CD56^{bright}$ $CD16^-$ NK cells	$CD45^+$ $CD3^-$ $CD56^{++}$ $CD16^-$	NK cells
$CD56^{low}$ $CD16^-$ NK cells	$CD45^+$ $CD3^-$ $CD56^+$ $CD16^-$	NK cells
$CD56^{low}$ $CD16^+$ NK cells	$CD45^+$ $CD3^-$ $CD56^+$ $CD16^+$	NK cells

**Myeloid Panel**		
Neutrophils	$CD45^+$ $CD16^+$	$CD45^+$ alive
Neutrophils $CD16^+$ $CD15^{low}$	$CD45^+$ $CD16^+$ $CD15^{low}$	Neutrophils
Neutrophils $CD16^+$ $CD15^+$	$CD45^+$ $CD16^+$ $CD15^+$	Neutrophils
Eosinophils	$CD45^+$ $CD16^-$ Siglec-8$^+$	$CD45^+$ alive
Atypical monocytes	SSC/FSC	$CD45^+$ alive
Monocytes	SSC/CD14+	$CD45^+$ alive
$CD14^+$ $CD16^-$ monocytes	$CD45^+$ $CD14^+$ $CD16^-$	Monocytes
$CD14^+$ $CD16^+$ monocytes	$CD45^+$ $CD14^+$ $CD16^+$	Monocytes
$CD14^{low}$ $CD16^+$ monocytes	$CD45^+$ $CD14^{low}CD16^+$	Monocytes

Table S2. Flow cytometry panels used to evaluate the study groups

Antibody cocktail	Fluorophore	Company and Reference number	Volume per test (uL)
**#1: Neutrophils, Eosinophils, monocytes, B, NK, NKT lymphocytes**			
CD45	APC-H7	BD. 560178	1.25
CD16	APC	Palex. 302012	0.63
CD15	BV510	BD. 563141	1.25
Siglec-8	BV711	BD. 747870	1.25
CD3	APC-R700	BD. 565119	0.63
CD19	BV421	BD. 562440	0.63
CD56	BV785	Palex. 362549	0.31
CD14	FITC	BD. 555397	10
CD163	PE-Cy7	Biolegend. 333614	2.5
**#2: T lymphocytes subpopulations**			
CD45	APC-H7	BD. 560178	1.25
CD3	APC-R700	BD. 565119	0.63
CD4	BV711	BD. 563028	2.5
CD8	BV650	BD. 563821	1.25
CD45RA	FITC	BD. 555488	1.25
CD197 (CCR7)	PE-CFS594	BD. 562381	2.5
CD196	PE-Cy7	BD. 560620	2.5
CD183 (CXCR3)	APC	BD. 550967	5
CD28	BV510	BD. 563075	1.25
CD25	PE	BD. 555432	10
CD127	BV786	BD. 563324	0.63
HLA-DR	BV421	BD. 562804	1.25
PD-1	PerCpCy5.5	Biolegend 329914	2.5

Table S3. Differential distribution of the immune populations assessed between age-smoking matched controls and IPF. A Saphiro test was performed for each variable and the appropriate statistic test was selected accordingly to their distribution using "compareGroups" R package. Data is shown as mean ± SD or median [IQR] accordingly.

Baseline immune populations	Control (n=32)	IPF (n=32)	p-value
**Innate immune cells**			
Eosinophils	0.77 [0.43;1.42]	1.56 [0.42;2.39]	0.260
Neutrophils	61.4 [54.0;63.9]	65.8 [63.2;69.0]	***0.001***
Monocytes	7.14 [6.19;7.81]	6.92 [5.99;7.44]	0.612
$CD14^+$ $CD16^-$ monocytes	87.7 [83.3;88.9]	88.1 [85.4;90.3]	0.349
$CD14^+$ $CD16^+$ monocytes	6.65 [5.80;9.31]	6.96 [5.28;9.74]	0.773

CD14^{low} CD16^{+} monocytes	3.09 [2.23;3.81]	2.74 [1.81;3.71]	0.272
NK cells	11.3 [7.21;15.6]	11.0 [6.95;16.4]	0.978
CD56^{bright} CD16^{-} NK cells	3.96 [2.55;6.84]	2.75 [1.88;4.89]	0.143
CD56^{dim} CD16^{+} NK cells	90.0 [84.6;91.8]	90.3 [84.1;94.5]	0.486
CD56^{dim} CD16^{-} NK cells	4.79 [3.92;8.02]	4.61 [2.05;8.23]	0.220
NKT cells	2.34 [1.40;5.54]	2.31 [1.46;4.08]	0.490
**Adaptive immune cells**			
Lymphocytes	29.6 [27.4;37.0]	25.1 [22.0;27.2]	<***0.001***
B cells	9.51 [7.51;11.0]	6.84 [4.98;10.7]	***0.025***
CD8^{+} T cells	31.4 (9.64)	32.8 (12.8)	0.644
CD8^{+} HLA-DR^{+} T cells	16.1 [12.4;22.2]	22.5 [16.6;33.7]	***0.023***
CD8^{+} CD28^{-} T cells	42.0 [23.9;65.0]	54.8 [49.2;69.6]	***0.040***
Effector CD8^{+} T cells	27.8 [17.3;38.8]	35.9 [15.9;46.2]	0.324
Central memory CD8^{+} T cells	15.4 [7.86;21.8]	8.87 [5.53;12.9]	***0.016***
Effector memory CD8^{+} T cells	39.8 (14.2)	46.9 (14.9)	0.055
Naive CD8^{+} T cells	9.34 [6.38;18.1]	6.48 [3.34;11.0]	***0.010***
CD4^{+} T cells	61.9 (11.1)	61.0 (14.2)	0.779
CD4^{+} HLA-DR^{+} T cells	8.77 [6.31;10.2]	9.08 [6.23;14.4]	0.229
CD4^{+} CD28^{-} T cells	3.58 [0.67;9.69]	3.46 [1.47;10.0]	0.814
Effector CD4^{+} T cells	0.32 [0.14;1.35]	0.53 [0.13;1.84]	0.643
Central memory CD4^{+} T cells	57.9 [50.5;63.6]	53.7 [42.7;65.8]	0.330
Effector memory CD4^{+} T cells	23.6 [16.2;30.7]	21.5 [15.2;34.0]	0.989
Naive CD4^{+} T cells	17.9 [12.3;20.7]	15.1 [11.4;27.3]	0.973
Th1 cells	15.5 (7.87)	23.1 (10.4)	***0.002***
Th17 cells	15.4 [10.8;20.9]	7.83 [6.32;12.8]	***<0.001***
Th1Th17 cells	6.42 [4.17;8.46]	3.96 [2.69;6.88]	***0.021***
Regulatory T cells (Tregs)	6.04 [5.25;7.87]	7.20 [5.85;9.03]	0.072
PD-1^{+} cells	1.96 [1.63;3.62]	2.82 [1.30;6.32]	0.679
**Ratios**			
NLR(Neutrophil-to-lymphocyte Ratio)	2.09 [1.45;2.27]	2.61 [2.40;3.05]	<0.001
MLR(Monocyte-to-lymphocyte Ratio)	0.23 [0.18;0.27]	0.26 [0.23;0.35]	0.012
CD4/CD8 Ratio	1.95 [1.42;2.80]	1.97 [1.27;2.92]	0.809
Th1/Th17 Ratio	0.97 [0.63;1.72]	3.14 [1.78;3.94]	<0.001
Th17/Tregs Ratio	2.46 [1.76;3.62]	1.09 [0.87;1.66]	***<0.001***

Table S4. Spearman correlations between baseline blood immune populations and inflammation ratios, and lung function at recruitment in IPF patients.

Baseline immune populations	Baseline FVC%		Baseline DLCO%	
	**Rho**	**p-value**	**Rho**	**p-value**
**Innate immune cells**				
Eosinophils	0,349	0,186	0,662	***0,010***
Neutrophils	-0,124	0,498	-0,496	***0,005***
Monocytes	0,074	0,688	0,112	0,557
$CD14^{+}$ $CD16^{-}$ monocytes	-0,041	0,822	-0,144	0,447
$CD14^{+}$ $CD16^{+}$ monocytes	0,136	0,458	0,028	0,884
$CD14^{low}$ $CD16^{+}$ monocytes	0,088	0,634	0,294	0,114
NK cells	0,037	0,840	-0,271	0,147
$CD56^{bright}$ $CD16^{-}$ NK cells	-0,102	0,578	0,138	0,467
$CD56^{dim}$ $CD16^{+}$ NK cells	-0,063	0,731	-0,152	0,422
$CD56^{dim}$ $CD16^{-}$ NK cells	0,141	0,443	0,175	0,354
NKT cells	-0,141	0,440	-0,123	0,517
**Adaptive immune cells**				
Lymphocytes	0,220	0,226	0,470	***0,009***
B cells	0,001	0,997	-0,091	0,631
$CD8^{+}$ T cells	-0,227	0,212	-0,213	0,259
$CD8^{+}$ HLA-DR^{+} T cells	-0,142	0,437	-0,005	0,981
$CD8^{+}$ $CD28^{-}$ T cells	-0,273	0,130	-0,193	0,306
Effector $CD8^{+}$ T cells	0,009	0,959	0,085	0,654
Central memory $CD8^{+}$ T cells	-0,132	0,473	-0,044	0,817
Effector memory $CD8^{+}$ T cells	-0,063	0,733	-0,186	0,326
Naive $CD8^{+}$ T cells	0,268	0,138	0,203	0,282
$CD4^{+}$ T cells	0,235	0,195	0,252	0,180
$CD4^{+}$ HLA-DR^{+} T cells	0,033	0,860	-0,064	0,738
$CD4^{+}$ $CD28^{-}$ T cells	-0,043	0,814	-0,149	0,431
Effector $CD4^{+}$ T cells	-0,063	0,733	0,049	0,798
Central memory $CD4^{+}$ T cells	-0,410	0,020	-0,272	0,146
Effector memory $CD4^{+}$ T cells	-0,184	0,312	-0,067	0,727
Naive $CD4^{+}$ T cells	0,533	***0,002***	0,192	0,309
Th1 cells	-0,116	0,528	-0,058	0,761
Th17 cells	-0,290	0,107	-0,215	0,255
Th1Th17 cells	-0,142	0,439	-0,056	0,770
Regulatory T cells (Tregs)	0,101	0,584	-0,176	0,353
PD-1^{+} cells	-0,121	0,510	-0,015	0,936
**Ratios**				
NLR(Neutrophil-to-lymphocyte Ratio)	-0,129	0,482	-0,467	***0,009***
MLR(Monocyte-to-lymphocyte Ratio)	-0,216	0,234	-0,434	***0,016***
CD4/CD8 Ratio	0,232	0,201	0,237	0,207
Th1/Th17 Ratio	0,205	0,260	0,137	0,472
Th17/Tregs Ratio	-0,293	0,103	-0,040	0,832

Table S5. Differential distribution of the baseline immune populations assessed between the two subgroups of IPF. Data is shown as mean ± SD or median [IQR]. A Saphiro test was performed for each variable and the appropriate statistic test was selected accordingly to their distribution.

**Baseline immune populations**	**Progressor (n=18)**	**Stable (n=13)**	**p-value**
**Innate immune cells**			
Eosinophils	0.98 [0.40;1.92]	1.96 [1.54;3.31]	0.278
Neutrophils	65.6 [63.4;66.2]	66.5 [55.8;69.9]	0.471
Monocytes	7.01 [6.04;7.94]	6.47 [5.61;7.12]	0.317
$CD14^{+}$ $CD16^{-}$ monocytes	87.5 [85.2;90.3]	88.2 [86.8;90.3]	0.734
$CD14^{+}$ $CD16^{+}$ monocytes	6.52 [5.11;11.4]	7.50 [5.68;9.20]	0.857
$CD14^{low}$ $CD16^{+}$ monocytes	2.74 [2.25;3.89]	2.86 [1.77;3.20]	1.000
NK cells	11.0 [6.07;16.0]	8.39 [7.16;16.3]	0.826
$CD56^{bright}$ $CD16^{-}$ NK cells	3.43 [1.92;5.60]	2.16 [1.99;3.87]	0.447
$CD56^{dim}$ $CD16^{+}$ NK cells	86.9 [82.4;94.4]	92.6 [87.9;94.5]	0.562
$CD56^{dim}$ $CD16^{-}$ NK cells	6.30 [2.10;10.4]	3.04 [1.95;4.94]	0.347
NKT cells	2.79 [2.06;5.07]	1.54 [0.92;2.79]	***0.031***
**Adaptive immune cells**			
Lymphocytes	25.4 [24.0;27.2]	24.9 [18.9;27.1]	0.734
B cells	6.49 [4.74;8.99]	9.13 [5.55;14.2]	0.186
$CD8^{+}$ T cells	39.4 (10.6)	22.4 (8.20)	***<0.001***
$CD8^{+}$ HLA-DR^{+} T cells	29.2 (15.3)	21.4 (13.5)	0.145
$CD8^{+}$ $CD28^{-}$ T cells	64.4 (12.0)	39.6 (18.0)	***<0.001***
Effector $CD8^{+}$ T cells	40.6 [25.9;58.0]	22.5 [14.3;42.1]	0.109
Central memory $CD8^{+}$ T cells	7.28 [4.68;10.7]	11.8 [8.42;16.5]	***0.020***
Effector memory $CD8^{+}$ T cells	44.9 [31.0;60.1]	42.1 [37.2;59.2]	0.435
Naive $CD8^{+}$ T cells	4.22 [2.30;7.79]	9.84 [6.33;12.6]	***0.024***
$CD4^{+}$ T cells	53.6 (11.2)	72.5 (9.80)	***<0.001***
$CD4^{+}$ HLA-DR^{+} T cells	11.6 [8.63;16.4]	7.58 [6.08;13.5]	0.128
$CD4^{+}$ $CD28^{-}$ T cells	4.31 [1.98;15.8]	1.62 [0.54;5.59]	***0.050***
Effector $CD4^{+}$ T cells	0.69 [0.18;2.17]	0.51 [0.11;1.61]	0.548
Central memory $CD4^{+}$ T cells	52.3 (14.7)	54.5 (13.8)	0.675
Effector memory $CD4^{+}$ T cells	30.0 (11.1)	16.3 (8.40)	***0.001***
Naive $CD4^{+}$ T cells	13.5 [10.3;15.3]	26.2 [14.7;39.6]	***0.013***
Th1 cells	26.1 (10.4)	19.0 (9.58)	0.061
Th17 cells	7.63 [5.03;12.1]	7.93 [6.90;14.7]	0.298
Th1Th17 cells	4.87 [2.13;7.29]	3.71 [3.07;5.00]	0.749
Regulatory T cells (Tregs)	7.50 (2.71)	7.40 (1.76)	0.902
PD-1^{+} cells	2.40 [1.31;3.94]	1.27 [0.94;2.15]	0.230
**Ratios**			
NLR(Neutrophil-to-lymphocyte Ratio)	2.51 [2.43;2.75]	2.67 [2.06;3.47]	0.575
MLR(Monocyte-to-lymphocyte Ratio)	0.27 [0.24;0.33]	0.24 [0.20;0.39]	0.496
CD4/CD8 Ratio	1.46 [0.99;2.03]	2.94 [2.76;4.22]	***<0.001***
Th1/Th17 Ratio	3.49 (1.99)	2.41 (1.70)	0.115
Th17/Tregs Ratio	1.04 [0.84;1.76]	1.11 [0.97;1.58]	0.401

Table S6: Differential distribution of the significant immune cell populations across groups (Controls, Stable and Progressors). Differences in the distribution were assessed using Kruskal-Wallis and post-hoc Mann-Whitney.

	Mean±SD			Kruskal-Wallis	Mann-whitney Post-hoc		
	Control	Stable	Progressor	p-value	Control vs Stable	Control vs Progressor	Progressor vs Stable
**Innate immune cells**							
Neutrophils	58,41±7,88	65,05±15,1	63,78±11,47	0,006	***0,048***	***0,010***	0,483
**Adaptive immune cells**							
Lymphocytes	30,14±7,31	24,54±12,83	24,51±7,64	0,010	***0,032***	***0,030***	0,904
B cells	9,96±2,78	11,4±8,74	7,98±5,5	0,036	0,615	***0,024***	0,294
$CD8^+$ T cells	31,44±9,64	22,44±8,2	39,42±10,6	0,000	***0,007***	***0,018***	***0,000***
$CD8^+$ $HLA\text{-}DR^+$ T cells	17,75±7,41	21,44±13,45	29,21±15,25	0,018	0,540	***0,014***	0,164
$CD8^+$ $CD28^-$ T cells	43,39±22,01	39,59±17,98	64,4±11,98	0,001	0,647	***0,002***	***0,002***
Central memory $CD8^+$ T cells	15,5±8,59	13,42±6,81	8,81±5,99	0,010	0,540	***0,012***	***0,032***
Naive $CD8^+$ T cells	14,47±13,32	10,9±7,44	5,74±4,6	0,003	0,647	***0,002***	***0,037***
$CD4^+$ T cells	61,91±11,09	72,52±9,79	53,58±11,2	0,000	***0,004***	***0,028***	***0,000***
Effector memory $CD4^+$ T cells	24,63±11,97	16,32±8,4	29,97±11,06	0,002	***0,019***	0,070	***0,005***
Naive $CD4^+$ T cells	18,94±11,95	27,33±15,86	14,17±7,53	0,023	0,088	0,088	***0,041***
Th1 cells	15,49±7,87	19±9,58	26,08±10,44	0,001	0,220	***0,001***	0,085
Th17 cells	17,29±9,24	12,32±9,13	9,29±5,24	0,001	***0,024***	***0,001***	0,312
NKT cells	4,23±3,91	2,03±1,38	4,75±5,38	0,072	0,087	0,562	0,087
**Ratios**							
NLR(Neutrophil-to-lymphocyte Ratio)	1,92±0,61	4,61±5,19	3,01±1,74	0,002	***0,031***	***0,001***	0,594
MLR(Monocyte-to-lymphocyte Ratio)	0,22±0,06	0,37±0,32	0,35±0,22	0,045	0,251	***0,047***	0,514
CD4/CD8 Ratio	2,35±1,52	4,07±2,99	1,53±0,72	0,000	***0,008***	***0,020***	***0,000***

Th1/Th17 Ratio	1,23±0,94	2,41±1,7	3,49±1,99	9,88E+09	***0,038***	***2,69E+09***	0,183
Th17/Tregs Ratio	2,83±1,64	2±2,43	1,29±0,67	0,000	***0,006***	***0,000***	0,417

Supplementary Figures

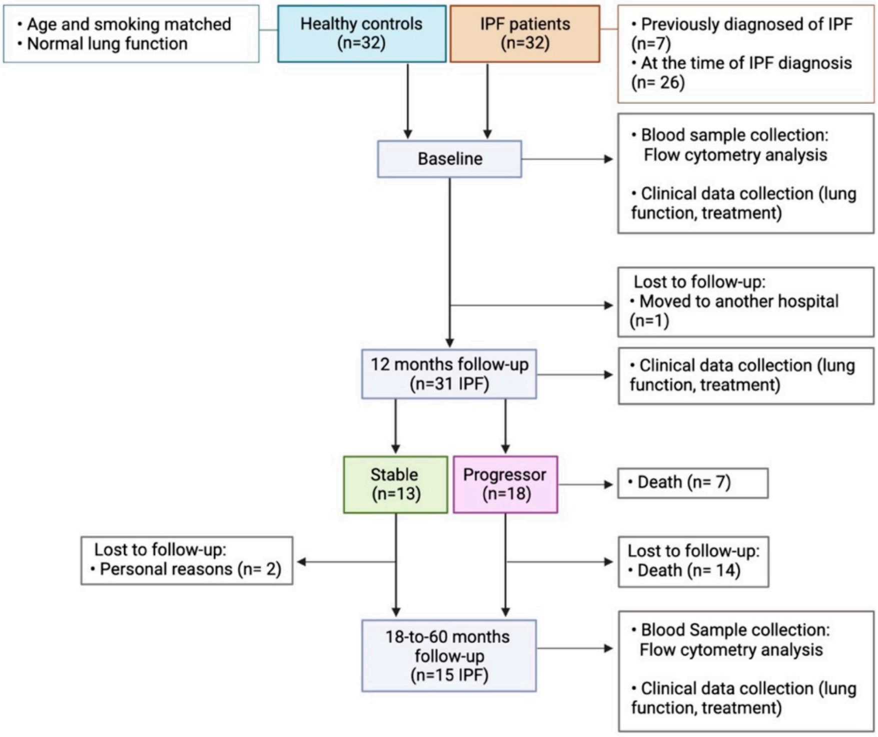

Figure S1. CONSORT flow diagram showing participant flow through each stage of the study (Created with BioRender.com).

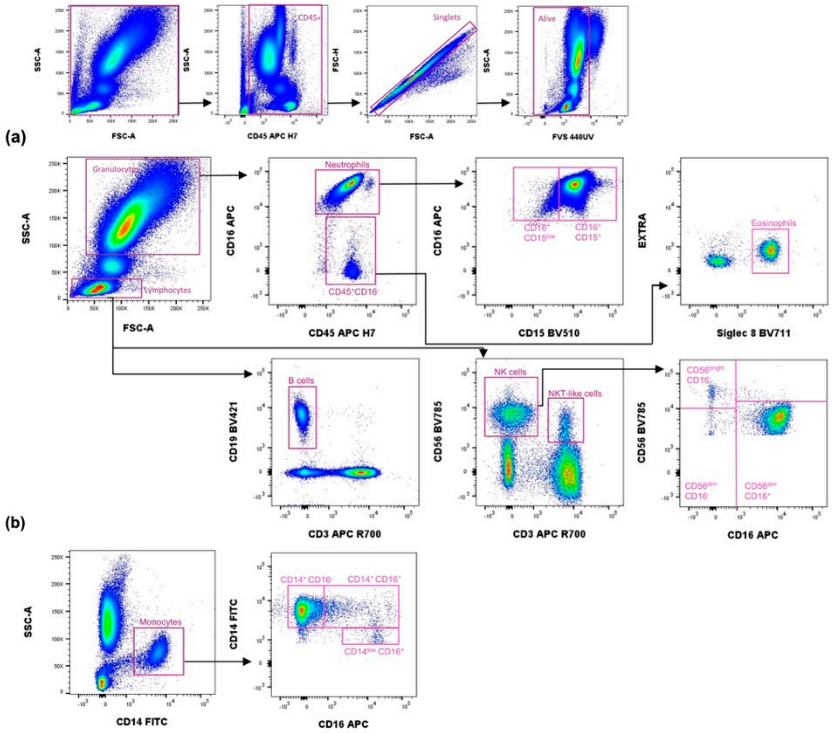

Figure S2. Representative gating strategy 1 for the evaluation of immune parameters in peripheral blood from IPF patients or healthy individuals. Flow cytometry staining was performed as described in *Methods.* The gating strategy outlined in A) shows immune panel used to identify the following populations from a sample after excluding debris and gating on $CD45^{+}$ single live cells: neutrophils, eosinophils, B cells, NK and NKT cell populations. Gating strategy B) shows monocytes and their subcategorization into classical ($CD14^{+}CD16^{-}$). intermediate ($CD14^{+}CD16^{+}$) and non-classical ($CD14^{low}CD16^{+}$).

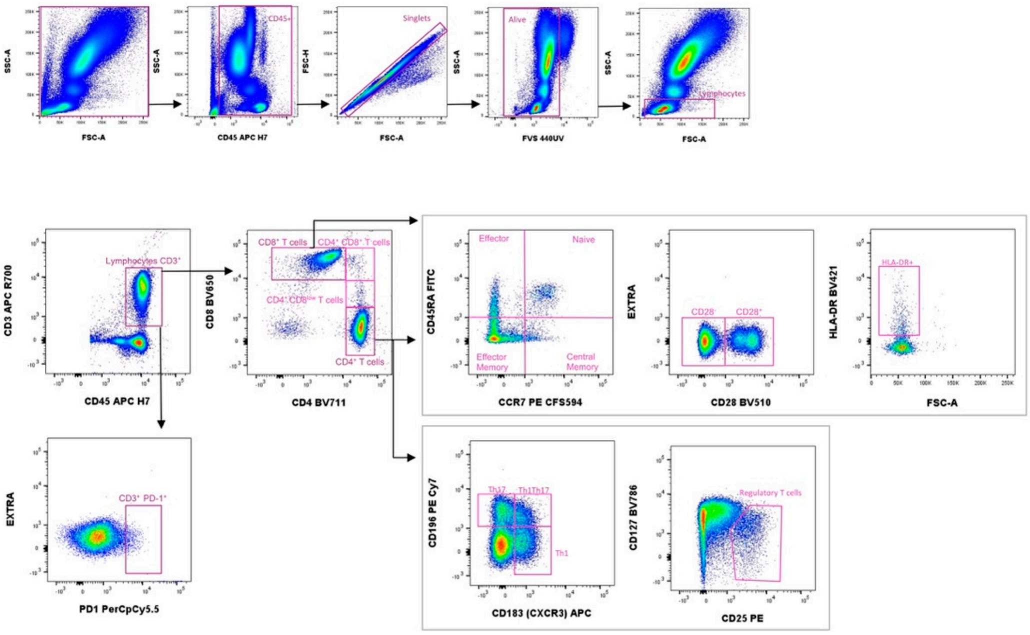

Figure S3. Representative gating strategy 2 for the evaluation of immune parameters in peripheral blood from IPF patients or healthy individuals. Flow cytometric staining was performed as described in *Methods.* The gating strategy shows immune panel used to identify the following T cell populations from a sample after excluding debris and gating on $CD45^{+}$ single live cells: $CD8^{+}$, $CD4^{+}$, Th1, Th17, Treg cells, Effector, Naive, Effector memory and Central memory T cells. Populations of $CD8^{+}$ and $CD4^{+}$ were also evaluated for the expression of CD28, PD-1 and HLA-DR activation and/or exhaustion markers.
